# Complement activation mediates cetuximab inhibition of non-small cell lung cancer tumor growth *in vivo*

**DOI:** 10.1186/1476-4598-9-139

**Published:** 2010-06-07

**Authors:** Yi-Fan Hsu, Daniel Ajona, Leticia Corrales, Jose M Lopez-Picazo, Alfonso Gurpide, Luis M Montuenga, Ruben Pio

**Affiliations:** 1 Division of Oncology, Center for Applied Medical Research (CIMA), Pamplona, Spain; 2 Department of Oncology, Clinica Universidad de Navarra, Pamplona, Spain; 3 Department of Histology, School of Medicine. University of Navarra, Pamplona, Spain; 4 Department of Biochemistry, School of Sciences. University of Navarra, Pamplona, Spain

## Abstract

**Background:**

Cetuximab, an antibody targeting the epidermal growth factor receptor (EGFR), increases survival in patients with advanced EGFR-positive non-small cell lung cancer when administrated in combination with chemotherapy. In this study, we investigated the role of complement activation in the antitumor mechanism of this therapeutic drug.

**Results:**

EGFR-expressing lung cancer cell lines were able to bind cetuximab and initiate complement activation by the classical pathway, irrespective of the mutational status of EGFR. This activation led to deposition of complement components and increase in complement-mediated cell death. The influence of complement activation on the activity of cetuximab *in vivo *was evaluated in xenografts of A549 lung cancer cells on nude mice. A549 cells express wild-type EGFR and have a KRAS mutation. Cetuximab activity against A549 xenografts was highly dependent on complement activation, since complement depletion completely abrogated the antitumor efficacy of cetuximab. Moreover, cetuximab activity was significantly higher on A549 cells in which a complement inhibitor, factor H, was genetically downregulated.

**Conclusions:**

We demonstrate for the first time that the *in vivo *antitumor activity of cetuximab can be associated with a complement-mediated immune response. These results may have important implications for the development of new cetuximab-based therapeutic strategies and for the identification of markers that predict clinical response.

## Background

Lung cancer accounts for more than 25% of all cancer deaths in United States [1]. Non-small cell lung cancer (NSCLC) represents about 80% of all lung cancers. Current treatment options consist of surgical resection, platinum-based doublet chemotherapy, and radiation. Unfortunately, despite these therapies, the prognosis remains poor. Recent advances in the understanding of the molecular pathogenesis of the disease have led to the development of molecular targeted therapies for NSCLC [2]. Bevacizumab, a monoclonal antibody to vascular endothelial growth factor, and erlotinib, a small-molecule tyrosine kinase inhibitor (TKI) of epidermal growth factor receptor (EGFR), are targeted agents approved in the treatment of NSCLC [3]. The clinical efficacy of cetuximab, a humanized monoclonal antibody against the extracellular domain of EGFR, has also been evaluated. A randomized phase III trial has recently shown significantly prolonged survival of advanced NSCLC patients who received cetuximab in combination with platinum-based chemotherapy as first-line treatment [4]. Conversely, combinations of gefitinib or erlotinib, EGFR tyrosine kinase inhibitors (TKIs), with standard chemotherapy in advanced NSCLC have failed to show clinical benefit [5-8]. Another remarkable observation is that, in contrast to the evidence for TKI treatment, KRAS mutation status does not appear to be predictive of response to cetuximab in NSCLC [9-11]. These data strongly suggest clinically relevant differences between the mechanisms of action of EGFR-TKIs and cetuximab [12]. In this sense, it has been suggested that immune mechanisms may contribute to the antitumor activity of cetuximab [13]. In particular, cetuximab, alone or in combination with other antibodies, may elicit immunological responses such as antibody-dependent cellular cytotoxicity (ADCC) or complement activation [14-17].

A better understanding of the mechanisms that govern cetuximab antitumor activity is necessary to optimize its therapeutic efficacy and to identify those patients who are going to benefit from the treatment. In the current report we investigated the influence of the activation of complement in the action of cetuximab in an *in vivo *animal model. We also explored the possibility of enhancing complement activation in an attempt to increase the clinical efficacy of cetuximab.

## Methods

### Lung cancer cell lines

A549 (lung adenocarcinoma), HCC827 (lung adenocarcinoma), and H187 (small-cell lung carcinoma) cell lines were obtained from the American Type Culture Collection. Cells were grown in RPMI 1640 supplemented with 10% Fetalclone III (Hyclone), 100 U/ml penicillin, and 100 μg/ml streptomycin.

### Sera

Normal human serum (NHS) was used as the source of complement. A pool of sera from ten healthy donors was prepared. Heat inactivated NHS (HI-NHS) was obtained by incubation of the serum at 56°C for 30 minutes.

### EGFR mRNA expression

RNA was purified from cells using the Ultraspec Total RNA Isolation Reagent (Biotecx). RNA was reverse transcribed and the expression of human EGFR mRNA was analyzed by PCR using the following primers: sense 5'-GGACGACGTGGTGGATGCCG-3', antisense 5'-GGCGCCTGTGGGGTCTGAGC-3'. GAPDH was used as an internal control. Primers for GAPDH mRNA amplification were: sense 5'-ACTTTGTCAAGCTCATTTCC-3', antisense 5'-CACAGGGTACTTTATTGATG-3'. PCR conditions were: 1 cycle of 2 min at 95°C, followed by 30 cycles of 30 sec at 95°C, 30 sec at 55°C, and 30 sec at 72°C, and finishing with 10 min at 72°C.

### KRAS mutations

Human KRAS codon 12 mutations were assessed by sequencing. Genomic DNA was subjected to PCR amplification with the following set of intronic primers: sense 5'-CGATACACGTCTGCAGTCAA-3', antisense 5'-GGTCCTGCACCAGTAATATGC-3'. The PCR products were sequenced using the Big Dye Terminator V1.1 Cycle Sequencing Kit (Applied Biosystems) according to the protocol supplied by the manufacturer.

### C1q fixation

A polystyrene 96-well plate was coated with 30 to 2000 ng of antibody per well in 100 μl of 50 mM sodium bicarbonate (pH 8.3) during one hour at room temperature. After washing, the plate was blocked overnight at 4°C with Tris-buffered saline (TBS) containing 1% bovine serum albumin, and 0.1% Tween 20. After washing, normal human serum, used as the source of C1q, was added in 100 μl of veronal buffer [1.8 mM barbital, 3.1 mM barbituric acid, 141 mM sodium chloride, 0.5 mM MgCl_2 _and 0.15 mM CaCl_2 _(pH 7.4)] and incubated for 30 min at 37°C. The plate was washed and the assay was developed with a rabbit anti-human C1q antibody (1:500; Dako), a goat anti-rabbit antibody coupled to horseradish peroxidase (1:1,000; Sigma-Aldrich) and O-phenylenediamine dihydrochloride (Sigma-Aldrich). A human IgG1 antibody (Sigma-Aldrich) was used as a positive control. The anti-factor H monoclonal antibody OX24, and heat inactivated NHS (56°C for 30 minutes) were used as negative controls. Cetuximab was kindly provided by Merck KGaA. OX24 was obtained as previously described [18].

### Binding of cetuximab and deposition of complement components

Cells were detached from culture dishes with trypsin/EDTA (Lonza), washed once, and resuspended in veronal buffer. Cells (2 × 10^5^) were mixed and incubated for 15 min at 37°C with NHS (diluted 1:5) and cetuximab (40 μg/ml). After washing, cells were incubated for 30 min at 4°C with the following antibodies: fluorescein isothiocyanate (FITC)-conjugated goat anti-human IgG (1:100; Sigma-Aldrich), rabbit anti-human C1q (1:100; Dako), FITC-conjugated goat anti-human C3 (1:100, ICN Biomedicals), or mouse anti-human C5b-9 (1:100; Dako). Secondary antibodies were goat anti-rabbit IgG labeled with Alexa-Fluor 488 (1:100; Invitrogen), or FITC-conjugated goat anti-mouse IgG (1:100; Invitrogen). Cells were analyzed by flow cytometry.

### Complement-mediated cell death

Complement-mediated cell death is associated with DNA fragmentation [19]. DNA fragmentation was evaluated using a method previously described [20]. In brief, 7 × 10^5 ^cells were resuspended in 0.2 ml of RPMI medium containing 30% NHS (or HI-NHS), and treated with 40 μg/ml of cetuximab for 24 hours at 37°C. Afterwards, cells were pelleted and fixed in 2 ml of cold 70% ethanol for 60 min at 4°C. Cells were centrifuged, washed twice with PBS, resuspended in 0.5 ml of PBS, and incubated with 10 μl of 1 mg/ml RNase A (Sigma) during 1 hour at 37°C. Finally, 5 μl of 1 mg/ml 7-aminoactinomycin D (Sigma) were added and, after incubation in the dark for 15 min at room temperature, cells were analyzed by flow cytometry. The percentage of DNA giving fluorescence below the G_1_/G_0 _peak was taken as measure of cell death.

### Cell proliferation assay

Cells (2,000 A459 cells/well or 4,000 HCC827 cells/well) were seeded in 96-well plates in RPMI medium supplemented with 10% FBS. Twenty-four hours later, cells were treated with cetuximab at different concentrations, and incubated for another 24 hours. Ten microliters of MTT (3-(4,5-dimethylthiazol-2-yl)-2,5-diphenyltetrazolium bromide) at 5 g/l (Sigma) was added to each well and incubated for 4 hours at 37°C. Later, 100 μl of dimethyl sulfoxide was added to each well to dissolve the MTT-formazan crystals. Absorbance was measured at 540 nm, with a reference filter at 690 nm, using a microplate reader.

### Xenograft model

Care of the animals was in accord with our institution guidelines. A549 cells (15 × 10^6^) were mixed 1:1 with Growth Factor Reduced Matrigel Matrix (BD Biosciences), and injected subcutaneously on the right flank of 4-6 week old female athymic nude mice (Harlan Laboratories, Italy). Athymic mice are immunodeficient and cannot develop a complete adaptive immune response, but have complement and NK cell activities. Tumor growth was measured every 2-3 days. Tumor volume was calculated using the formula: Volume = length × width^2 ^× 0.5. Tumors were allowed to reach about 200-250 mm^3 ^before randomization. When indicated, complement was depleted with cobra venom factor (CVF), as previously described [21].

### C3 immunofluorescence

Xenografts were harvested, fixed in buffered formalin, paraffin-embedded and sectioned (5 μm thick). Slides were deparaffinized, blocked with normal rabbit serum (1:20 dilution), and incubated with a goat anti-mouse C3 (1:500; Santa Cruz Biotechnology). Afterwards, slides were washed and incubated with a rabbit anti-goat IgG coupled to Alexa-Fluor 488 (1:500; Invitrogen). Slides were washed, mounted, and analyzed in an Olympus fluorescence microscope.

### Statistical analysis

Data were analyzed by Student's t-test. A p value of less than 0.05 was considered as statistically significant.

## Results and Discussion

**Figure 1:**
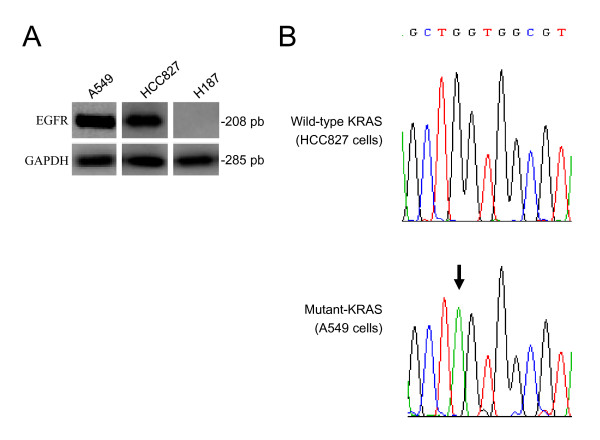
**Characterization of the lung cancer cell lines used in the study**. A, Expression of EGFR mRNA in A549, HCC827 and H187 cells determined by RT-PCR. GAPDH was used as a control. B, Confirmation of a KRAS codon 12 mutation (GGT > AGT) in A549 cells.

**Figure 2:**
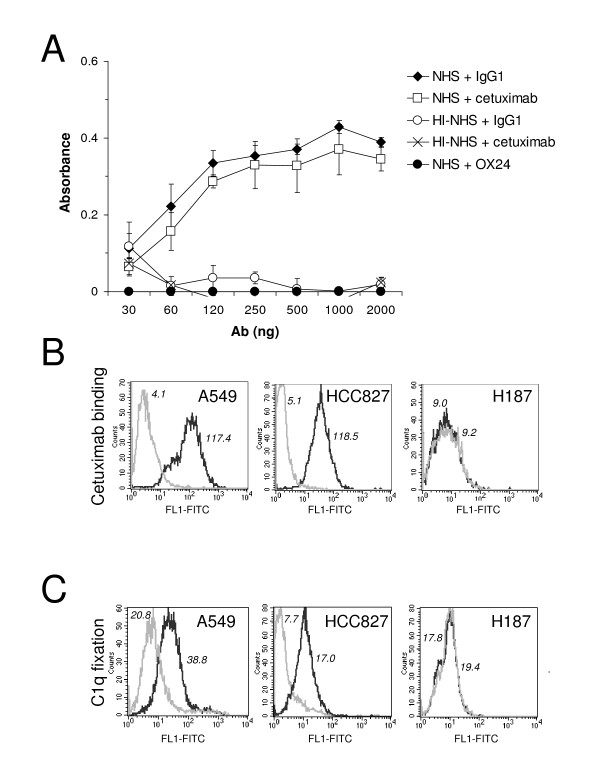
**Complement fixation by cetuximab**. A, C1q binding to cetuximab was first evaluated in wells coated with different amounts of antibody and incubated with 1% NHS. A human IgG1 antibody able to fix C1q was used as a positive control. Monoclonal antibody OX24 and heat inactivated NHS (HI-NHS) were used as negative controls. Data shows mean ± SEM. B, Flow cytometry analysis of the binding of cetuximab to lung cancer cells (black line). A human IgG1 was used as an isotype control (grey line). C, Lung cancer cells were incubated with NHS and cetuximab. The binding of C1q to the Fc-region of cetuximab was analyzed by flow cytometry with the anti-human C1q antibody (black line) or an isotype control (grey line). Mean fluorescence intensities (MFI) are indicated.

**Figure 3:**
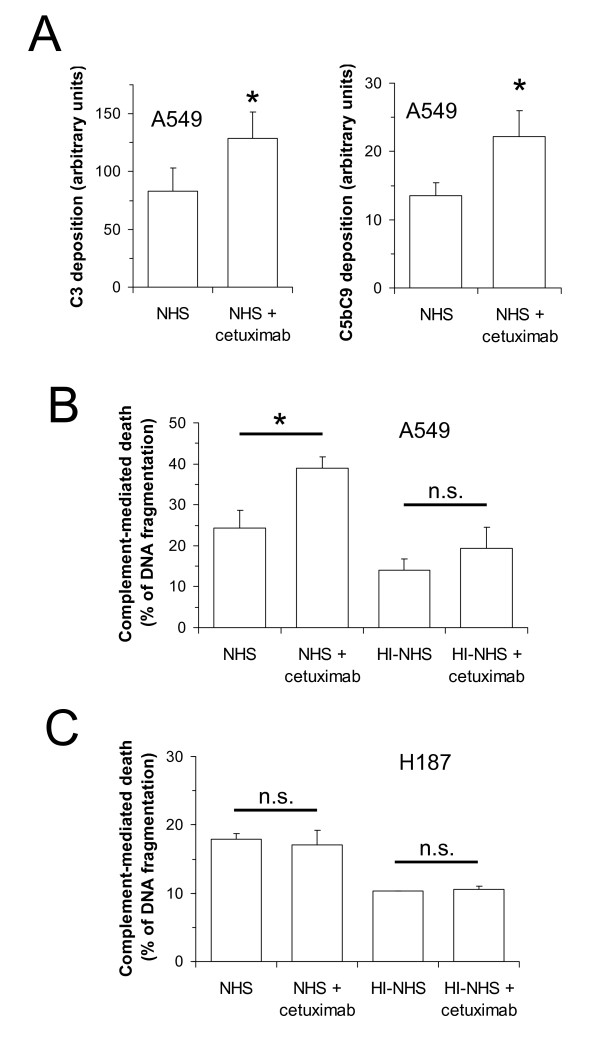
**Complement activation by cetuximab in lung cancer cells**. A, Deposition of C3-related fragments and C5b-C9 on A549 cells after incubation with NHS and cetuximab was determined by flow cytometry. Incubation with a human IgG1 isotype control was used as control. Data were collected as mean fluorescence intensity and the graphs show mean + SEM (*, p < 0.05). B and C, Complement-mediated cell death in A549 (B) and H187 (C) cells treated with cetuximab (40 μg/ml) and evaluated by flow cytometry. Data were collected as percentage of DNA fragmentation and the graph shows mean + SEM (*, *P *< 0.05; n.s., non-significant).

We used three different lung cancer cell lines in the study: A549, HCC827, and H187 cells. A549 cells express wild-type EGFR and have a KRAS mutation (Fig. 1A-B). HCC827 cells express mutated-EGFR and wild-type KRAS. H187 cells do not have detectable levels of EGFR. We first analyzed the capacity of cetuximab to fix complement. C1q from NHS was able to bind to wells coated with cetuximab (Fig. 2A), but not to wells coated with OX24, an IgG1 monoclonal antibody unable to fix complement [18]. C1q binding to cetuximab was inhibited by heat inactivation. We next assessed the capacity of cetuximab to fix complement on the membrane of lung cancer cells. We used 40 μg/ml of cetuximab (at the FDA-approved dosing level, according to the manufacturer, the peak and trough plasma concentrations of cetuximab range from 168 to 235 μg/ml and from 41 to 85 μg/ml, respectively). In cells expressing EGFR, cetuximab treatment initiated complement activation, irrespective of the mutational status of EGFR (Fig. 2B-C). Complement activation by the antibody-dependent classical pathway results in C3b-deposition, which leads to the formation of the cytolytic C5b-C9 complex [22]. Treatment of EGFR-expressing cells with cetuximab and NHS led to the deposition of these complement components and to complement-mediated cell death (Fig. 3A-B). In contrast, Kimura et al previously reported that complement dependent cytotoxicity was not detected in A549 cells treated with cetuximab [14], and Dechant et al found that complement activation only occurred when cetuximab was combined with other EGFR antibodies [17]. In both studies cetuximab was used at 10 μg/ml. We confirmed that there is not complement-mediated cell death with 10 μg/ml of cetuximab, but higher concentrations are required (data not shown). As mentioned above, the concentration used in the present study (40 μg/ml) is similar to the lowest concentration found in plasma from patients treated with the standard dosing of cetuximab. Treatment of EGFR-negative H187 cells with 40 μg/ml of cetuximab did not affect C3b-deposition, C5b-C9 complex formation, or complement-mediated cell death (Fig. 3C). These results suggest that, upon binding of cetuximab to the cell membrane of EGFR-positive cells, the classical pathway of complement is initiated.

**Figure 4:**
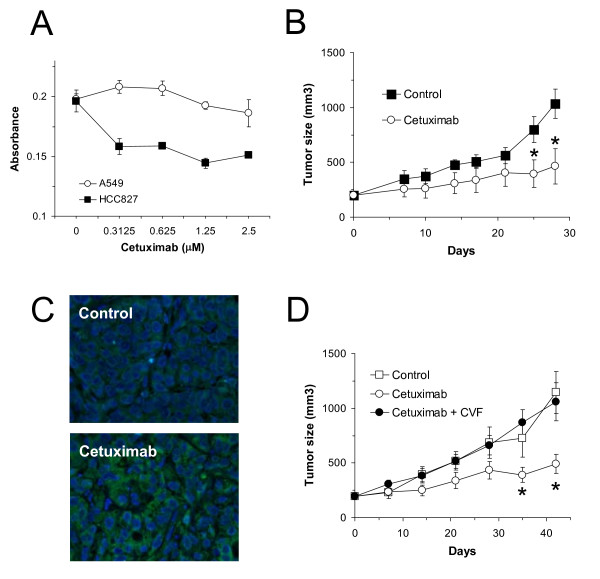
**Complement-dependent antitumor activity of cetuximab in A549 xenografts**. A, MTT assay for the *in vitro *antitumor activity of cetuximab in A549 and HCC827 lung cancer cell lines (with wild-type and mutant-EGFR, respectively). Cell proliferation was determined after 24 h treatment using an MTT assay. The graph shows mean ± SEM. B, A549 cells were injected s.c. on athymic nude mice. When tumors reached 200-250 mm^3^, mice (n = 10) were randomized into two groups: one was treated with cetuximab and the other with PBS. Cetuximab was administered i.p. at 0.5 mg per mouse three times a week and tumor growth was measured every 2-3 days. Data represent mean ± SEM (*, *P *< 0.05). C, Representative example of the deposition of C3-related fragments, determined by immunofluorescence, in explanted tumors from each experimental group. D, A549 xenografts were generated as above. Animals (n = 18) were divided into three groups. In one group, complement was depleted with CVF. Cetuximab treatment was administrated to the CVF-treated group and to a second group with full complement activity. The last group received PBS. Data represent mean ± SEM (*, *P *< 0.05).

It is important to realize that lysis is not the only biological consequence of complement activation *in vivo*. For example, complement activation releases anaphylatoxins that promote proinflammatory responses. In addition, the interaction of iC3b with CR3 (CD11b/CD18) on immune cells mediates complement-dependent cellular cytotoxicity (CDCC). Therefore, cetuximab-mediated complement activation may generate a powerful antitumor response in an *in vivo *setting. To test this hypothesis, we used A549 cells, which express wild-type EGFR and have a KRAS mutation. In *in vitro *studies, A549 cells are insensitive to the blockade of EGFR signaling and resistant to cetuximab [23]. This resistance may be a consequence of the presence of the KRAS mutation. However, cetuximab has some antitumor effect *in vivo *in A549 xenografts grown in nude mice [24]. This observation strongly suggests that cetuximab operates by *in vivo *mechanisms of action other than inhibition of EGFR-signaling. We first confirmed the resistance of A549 cells to cetuximab using an MTT assay (Fig 4A). Afterwards, we grew A549 xenografts in athymic mice and treated them with cetuximab. As previously reported [24], tumor growth was partially inhibited by cetuximab (Fig. 4B). Interestingly, explanted tumors from mice treated with cetuximab had high levels of C3 deposition, demonstrating the capacity of cetuximab to activate complement *in vivo *(Fig. 4C). The contribution of complement in the reduction of tumor growth was confirmed using complement-deficient mice. Complement depletion was achieved with cobra venom factor (CVF). Serum C3 levels were reduced to less than 10% as evaluated by ELISA (data not shown). Complement depletion completely abrogated the activity of cetuximab in A549 xenografts (Fig. 4D).

**Figure 5:**
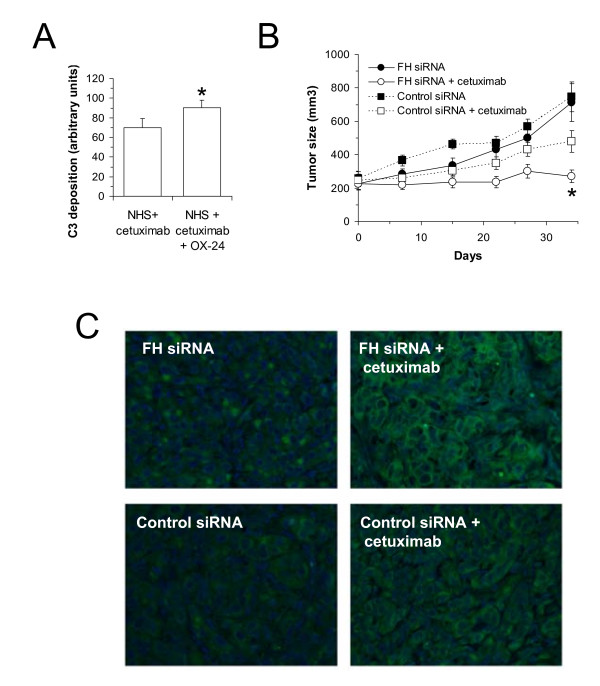
**Effect of factor H downregulation on cetuximab-mediated complement activation**. *A*, A549 cells were incubated with NHS and cetuximab in the presence or absence of OX24, an antibody that inhibits the binding of factor H to surface-bound C3b. Deposition of C3-related fragments, evaluated by flow cytometry, significantly increased in the presence of OX24 (*, *P *< 0.05). Data represent mean + SEM. *B*, Xenografts of A549 cells deficient in factor H (FH siRNA) were grown in nude mice until they reached ~250 mm^3 ^(n = 8). Half of the mice were treated with cetuximab, and the other half with PBS. The same experiment was conducted in A549 cells stably transfected with a scramble siRNA (control siRNA). Results shown represent mean ± SEM. The antitumor activity of cetuximab was significantly higher in factor H-deficient cells than in cells transfected with a control siRNA (*, *P *< 0.05). *C*, representative examples of the deposition of C3-related fragments in the explanted tumors.

The efficacy of complement-activating antibodies could be potentiated by the induction of immune effectors suppressed by the tumor microenvironment. In this line, we tried to increase the efficacy of cetuximab by eliminating the control exerted by factor H, a complement inhibitor that prevents an efficient immune response against lung cancer cells [21]. Incubation of A549 cells with NHS and cetuximab in the presence of OX24 significantly increased the deposition of C3-related fragments (Fig. 5A). OX24 is an antibody that inhibits the binding of factor H to surface-bound C3b. To evaluate the contribution of factor H to the control of complement-mediated cetuximab activity, we used A549 cells in which the expression of factor H was stably downregulated by siRNA [21]. The antitumor activity of cetuximab was significantly higher in factor H-deficient cells than in cells transfected with a control siRNA (Fig. 5B). Cetuximab-mediated tumor growth reduction was more than 90% in factor H-deficient cells, compared to around 50% in control cells. *In vivo *antitumor activity of cetuximab was again associated with an increase in the deposition of C3-related fragments (Fig. 5C). In conclusion, these results demonstrate for the first time that a complement-mediated immune response induced by cetuximab participates in the control of tumor cell growth *in vivo*. This mechanism of action provides numerous opportunities to enhance the efficacy of therapeutical monoclonal antibodies [25].

**Figure 6:**
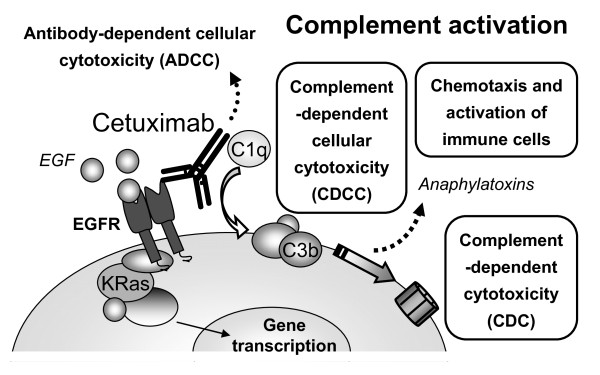
**Effector mechanisms triggered by cetuximab**. Cetuximab can inhibit EGFR-intracellular signaling or induce an immune response. These two antitumor mechanisms may act in conjunction. In certain conditions, such as in the presence of KRAS mutations, the immune response may be particularly important. The activation of the immune system by cetuximab can lead to both antibody-dependent cellular cytotoxicity (ADCC) and complement activation. Complement activation results in complement-dependent cellular cytotoxicity (CDCC) and/or complement-dependent cytotoxicity (CDC). CDCC is mediated by the interaction of C3b and its receptors in effector cells (e.i. phagocytes and NK cells). The release of anaphylatoxins, such as C5a, would further increase the recruitment and activation of effector cells. The three main immune consequences of complement activation are summarized in the boxes.

Human tumors in immunocompromised mouse models do not entirely behave as syngeneic tumors in immunocompetent hosts. For example, although athymic mice have normal complement activity, they cannot develop an adequate adaptive immune response. In an intact immune system, both ADCC and CDCC would likely be important mediators of cetuximab antitumor activity (Fig. 6). Thus, the efficacy of cetuximab against human tumors might be attenuated in our immunocompromised animal model. Still, our data clearly indicate that complement activation is sufficient to elicit an intense response able to induce more than 90%-reduction in the tumor growth of cells resistant to blockade of the EGFR-signaling pathway. It would be also possible that the activation of complement may contribute to the antitumor effect of cetuximab in cells sensitive to the blockade of this pathway.

It would be interesting to evaluate the immune mechanisms through which complement activation is able to control tumor growth. A direct effect of complement on the target cells may be accompanied by the recruitment and activation of cellular effectors such as lymphokine activated killer (LAK) cells and natural killer (NK) cells. In addition, the combination of cetuximab with modifiers of the immune response may be considered an attractive therapeutic approach to enhance the clinical efficacy of cetuximab against lung, colorectal, and head and neck carcinomas. For example, clinical trials are underway using cetuximab in combination with β-glucans to treat colorectal cancer. β-glucans are polysaccharides that collaborate with complement-activating antibodies in the elimination of tumors [26]. Cetuximab may also be combined with other monoclonal antibodies to potentiate complement activation [17]. Modifications in the Fc-region of the monoclonal antibody can also be attempted to increase the ability of the antibody's activation of complement [27].

In an unselected population, cetuximab treatment, as many other biological agents, only benefits a small percentage of NSCLC patients [28]. The body of knowledge that has paved the way for more targeted use of cetuximab has been recently reviewed [29]. Many groups are also focusing their research on markers of response (or resistance) to cetuximab. A more rational use of EGFR-targeted agents, such as cetuximab, should provide benefits for patients and health-care providers alike by sparing patients unnecessary treatment and allowing better use of health-care resources [29]. However, at present, no molecular biomarker is available to predict response to cetuximab in NSCLC. Based on our results, polymorphisms or other genetic traits that modulate the immune function may condition the activity of cetuximab, serving as markers to predict clinical response.

## Conclusions

There is a pressing need to elucidate the molecular mechanisms involved in cetuximab activity in order to improve its clinical efficacy and to better select patients who would benefit from cetuximab treatment. In this paper we demonstrate that a complement-mediated immune response participates in the inhibition of tumor cell growth by cetuximab *in vivo*. This observation may have important clinical implications in the development of new cetuximab-based therapeutic strategies and in the identification of markers that predict clinical response.

## Competing interests

The authors declare that they have no competing interests.

## Authors' contributions

JMLP, AG, LMM and RP conceived the study. YFH, LMM and RP were responsible for the experimental design. YFH, DA and LC performed experiments and helped in the analysis and interpretation. YFH, JMLP, AG, LMM and RP prepared the manuscript. All authors read and approved the final version of the manuscript.
